# Rehospitalizations for ambulatory care sensitive conditions in sepsis survivors– a nationwide cohort study using health claims data 2016–2019

**DOI:** 10.1007/s15010-025-02606-9

**Published:** 2025-07-18

**Authors:** Lisa Wedekind, Norman Rose, Antje Freytag, Aurelia Kimmig, Peter Schlattmann, Mathias W. Pletz, Thomas Ruhnke, Patrik Dröge, Carolin Fleischmann-Struzek

**Affiliations:** 1 Institute of Medical Statistics, Computer and Data Sciences, Jena University Hospital, Jena, Germany; 2 Institute of Infectious Diseases and Infection Control, Jena University Hospital, Jena, Germany; 3 Institute of General Practice and Family Medicine, Jena University Hospital, Jena, Germany; 4 AOK Research Institute (WIdO), Berlin, Germany

## Abstract

**Purpose:**

Sepsis survivors suffer from frequent rehospitalizations, of which a certain proportion is considered preventable by timely and adequate management in the outpatient setting (= ambulatory care sensitive conditions, ACSC). We aimed to assess the frequency of and risk factors for ACSC and infection-associated ACSC rehospitalization among sepsis survivors.

**Methods:**

Population-based, retrospective cohort study among using nationwide health claims data of the “AOK– die Gesundheitskasse”. Sepsis patients with inpatient treatment in 2016–2019 were identified using ICD-codes. Among sepsis hospital survivors, ACSC and infection-related ACSC were identified. Patient-related risk factors for ACSC were assessed by a multiple logistic regression analysis.

**Results:**

We included 347,826 sepsis patients and 234,874 sepsis hospital survivors. A total of 53.2% and 21.3% of sepsis survivors had at least one ACSC and infection-related ACSC rehospitalizations in the 12-months post-discharge, respectively. ACSC rehospitalizations often occurred closely after discharge and more frequently affected older, male, care dependent patients as well as those living in rural areas.

**Conclusion:**

ACSC are common among sepsis survivors. This underlines to need for structured aftercare programs and interventions in these patients, particularly for ACSC risk groups which comprise older, male, care dependent patients in rural areas.

## Introduction

Sepsis survivors are prone to post-acute, chronic health deteriorations [1]. They are more frequently hospitalized than patients after other acute medical conditions, often for recurrent infections and cardiovascular diseases [2]. A considerable proportion of rehospitalizations is caused by so-called ambulatory care sensitive conditions [3] (ACSC). The term ACSC defines health impairments, for which timely and adequate management in the outpatient setting could potentially prevent hospitalization. The concept of ACSCs emerged in the 1990s as part of efforts to evaluate the performance of health systems [4]. They comprise a set of both chronic (e.g., diabetes mellitus, congestive heart failure, hypertension, COPD) and acute conditions (e.g., urinary tract infections, dehydration, bacterial pneumonia), for which high-quality ambulatory care may prevent disease exacerbations, reduce complications, or avoid the need for hospital admission [5]. As such, hospitalization for an ACSC is considered, though not always definitively, a proxy for ambulatory care performance [6]. 

Rates of ACSC hospitalizations are typically elevated among vulnerable populations such as the elderly, individuals with low socioeconomic status, and patients with multiple chronic conditions [7, 8]. In the context of sepsis survivorship, this concept is particularly relevant. Sepsis survivors often face long-term physical, cognitive, and psychological impairments, as well as increased risk of chronic illness exacerbation and recurrent infections [1, 9]. The high rate of hospital readmissions in this population suggests that improved ambulatory care coordination, follow-up, and early intervention could contribute to mitigate avoidable rehospitalizations for ACSCs.

However, knowledge on the occurrence of ACSC rehospitalizations in sepsis survivors is scarce and risk groups for ACSC remain unknown, which hampers the design and implementation of preventive measures in this vulnerable patient cohort, e.g. by targeted aftercare programs, vaccination campaigns or regular monitoring on health detoriation post-discharge. Therefore, this study examines ACSC rehospitalizations in a population-based cohort of sepsis survivors within 12 months post-discharge.

## Methods

This retrospective cohort study forms part of the AVENIR study with is described in detail elsewhere [10]. We used nationwide health claims data of the German health insurance “AOK– Die Gesundheitskasse”, covering 27 million beneficiaries (32% of the German population). Patients ≥ 16 years with sepsis hospitalization between 2016 and 2019 were identified by ICD-10-codes for sepsis (explicit codes) and ICD-10 or procedural codes for organ dysfunction [10], who survived the hospitalization. Until 2020, ICD-10 codes for sepsis were still defined according to the sepsis-1/2 definition [11] in Germany. Therefore, we included codes for sepsis with organ dysfunction (R65.1 or R57.2) or combined sepsis codes with codes for organ dysfunction. We excluded patients without continuous insurance 12 months pre- and 24 months post-sepsis. After discharge, we analyzed an individual 12-months follow-up period dataset on hospital admissions. Outcomes were the frequency of rehospitalizations in the 12 months post-discharge with ACSC, and infection-associated ACSC. ACSC were defined based on previous literature, among which infection-related ACSC codes were selected [2, 3]. These comprised, among others, infections of the skin and subcutis, sexually transmitted diseases, Influenza and pneumonia, and vaccine-preventable diseases. Risk factors for ACSC rehospitalizations were assessed by multiple logistic regression analyses considering age, sex, place of residence (urban, rural as defined according to settlement structures defined by the German Federal Institute for Research on Building, Urban Affairs and Spatial Development [12], note that urban and suburban regions were summarized as urban regions) and nursing care dependency as predictors. Furthermore, we compared long-term mortality of patients with and without ACSC rehospitalization.

## Results

**Fig. 1:**
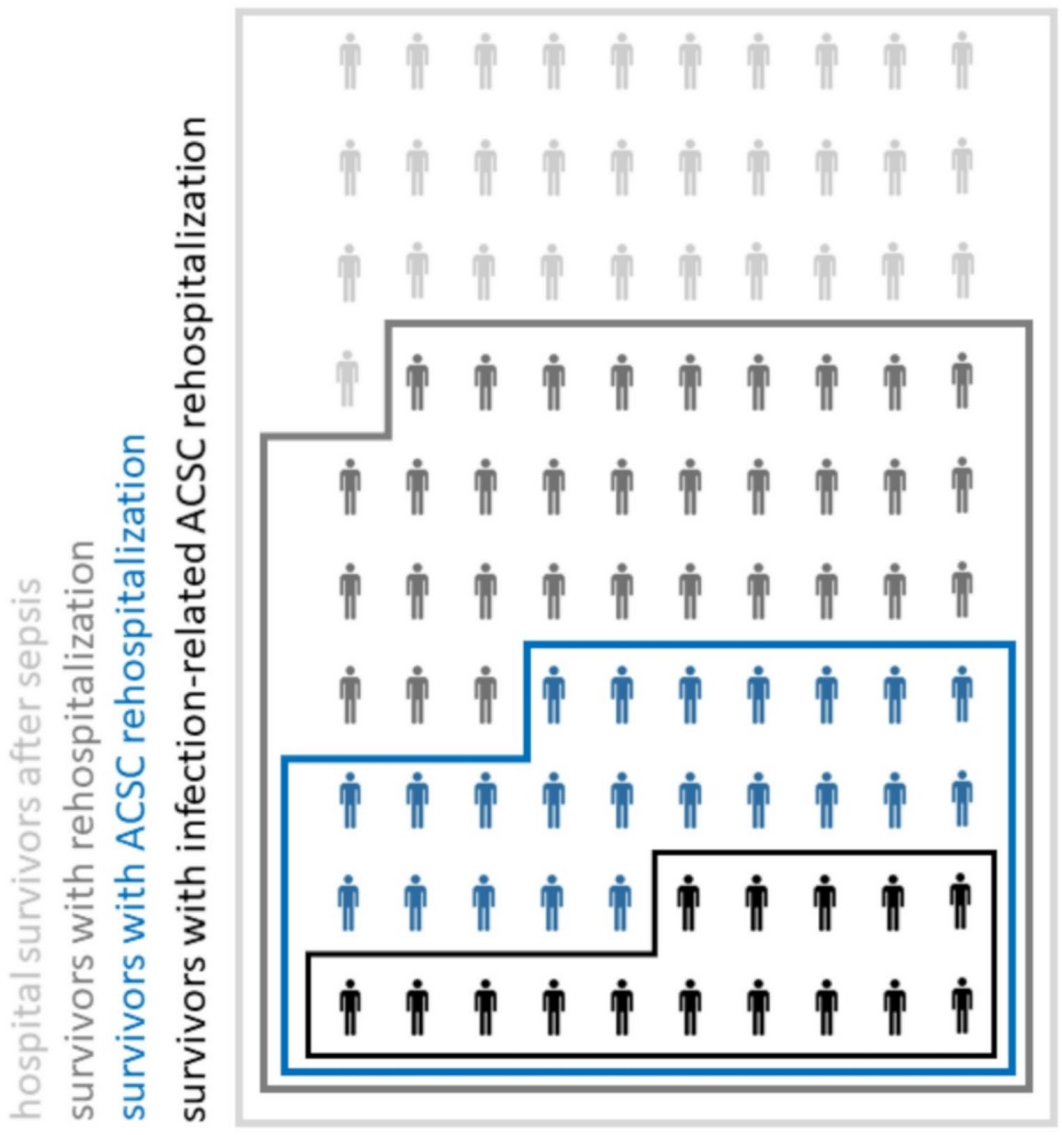
Types of rehospitalizations among sepsis survivors. Of 100 hospital survivors after sepsis (light gray), 70 had at least one rehospitalization in the 12 months post-sepsis (dark gray). 37 had a rehospitalization with ambulatory-care sensitive conditions (ACSC, blue) and 15 had an infection-related ACSC rehospitalizations (black)

**Fig. 2:**
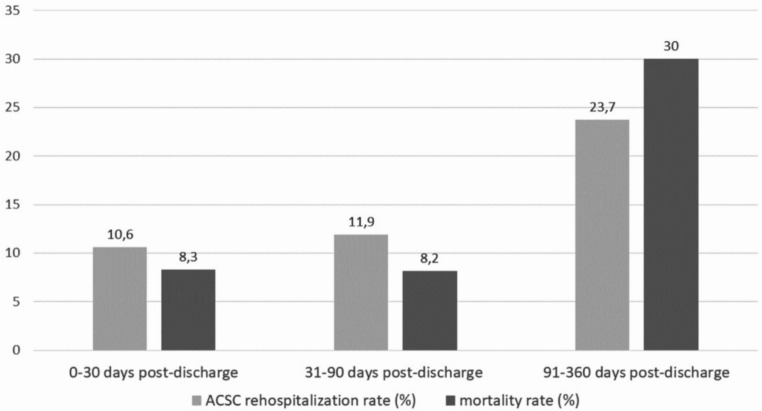
ACSC rehospitalization rate and mortality rate of patients in the three time frames (0–30 days, 31–90 days and 91–360 days post-discharge)

Among 23 million beneficiaries, we identified 347,826 sepsis patients and 234,874 sepsis hospital survivors (67.5%) between 2016 and 2019. Demographics and clinical features of sepsis hospital survivors are presented in Table 1. In the 12 months post-discharge, 68.9% (*n* = 161,828)of survivors had at least one rehospitalization, of which 53.2% (*n* = 86,092) were rehospitalised with ACSC and 21.3% (*n* = 34,469) with infection-associated ACSC respectively. Related to the total survivor population, ACSC and infection-related ACSC rehospitalizations affected 36.6% of hospital survivors and 14.7% of 12-months survivors post-discharge, respectively (Fig. 1). In the 12-months post-discharge, 108,675 ACSC rehospitalizations occurred. Many ACSC rehospitalization occurred early after discharge and thus in the time frame with a high mortality (Fig. 2). Higher age, male sex, nursing care dependency and rural place of residence were positively associated with the risk for ACSC both in the simple and multiple regression analyses (Table 2). Considering all other predictors, higher age increase the risk of ACSC rehospitalization between 1.6-fold (95% CI: 1.5; 1.7, age group 40–64) and 2 fold (95% CI: 1.9; 2.1, age group 65–79) compared to the reference age group of < 40 years. Nursing care dependency without nursing home led to an increase the risk by 1.6-fold compared to he reference of no nursing care dependency (95% CI: 1.6; 1.6). Rural residence was associated with a slight increase in the odds for ACSC rehospitalization (1.03 (95% CI: 1.01; 1.05)). Patients with ACSC rehospitalization had a higher 12-months mortality compared to patients without ACSC rehospitalization (31.4% vs. 29.3%, *p* < 0.001).

## Discussion

In this first study on ACSC rehospitalizations after sepsis in Germany, we found that one third of sepsis survivors is affected by ACSC rehospitalizations in the 12-months post-discharge. ACSC rehospitalizations often occur closely after discharge and more frequently affect older, male, care dependent patients as well as those living in rural areas. 21.3% of ACSC are infection-related and therefore may be partially vaccine-preventable, which can offer important targets for preventive measures to reduce the burden of sepsis.

Our ACSC rehospitalization rates are comparable with the proportion of ACSC rehospitalizations in sepsis found in a US study among veterans aged 65 years and above (37% in our study vs. 42% in the US), but higher than among general ICU survivors in the US (24% of rehospitalizations) [13]. The ACSC proportion among hospitalizations is also higher than estimated in the general German population (27% in 2012), which may underline the vulnerability of sepsis survivors in terms of recurrent infection and sepsis, but also cardiovascular and pulmonal diseases of which many are considered ambulatory-care sensitive.

In our studies, risk groups for ACSC rehospitalizations comprised older, male, care dependent patients and those living in rural communities. Intestingly, the odds for ACSC rehospitalization were lower in nursing home residents than in care dependent patients living in the community compared to a reference of non-care dependent patients. We hypothesize that this may be due to the fact that in Germany, each nursing home is typically affiliated with a designated general practitioner (GP) who is responsible for providing primary medical care to the residents. While residents formally retain the right to choose their own physician, in practice, many receive care from the nursing home’s assigned GP to ensure continuity and better coordination of care. This model facilitates close collaboration between medical staff and nursing personnel and supports timely medical interventions within the facility, which may contribute to a better prevention of ACSC rehospitalizations compared to patients with nursing care degree receiving care outside nursing homes. In this regard, a nationwide German study observed that nursing-home residents had significantly more contacts with general practitioners, medical specialists, and prescriptions for medical aids than their home-dwelling counterparts [14]. In rural patients, the higher ACSC risk may be driven by limited access to primary care and specialist services, longer travel distances to healthcare facilities and limited availability of public transportation [15], and an increasing shortage of healthcare professionals in these regions [16]. 

The high ACSC rehospitalization rates we observed, particularly closely after discharge, underscore the importance of seamless follow-up care in sepsis survivors, with a focus on physical rehabilitation, structured (outpatient) aftercare and social support measures for patients and their families [17, 18], and tailored to align with both the available resources and the living environments risk groups. To this end, a transitional care intervention with focus on care coordination, including screening on sepsis sequelae, management of chronic diseases and advance care planning was effective in reducing a composite endpoint of rehospitalization and mortality in sepsis survivors in a recent US randomized control trial [19]. Furthermore, immunization gaps should be closed and thereby can contribute to the prevention of infection-related ACSC, but also cardiovascular sequelae [20]. However, it also has to be acknowledged that only a certain proportion of ACSC rehospitalizations may be preventable by improved (ambulantory) aftercare. Therefore, the impact of aftercare interventions on ACSC rehospitalization rates and their preventability needs also be evaluated in further studies.

### Limitations

We used health claims data that are collected for reimbursement purposes and therefore may be influenced by external incentives within the DRG system. In this data, sepsis may be identified only with low sensitivity, and cases with lower severity may be missed [21]. This may similarly apply for ACSC, although we lack data on the validity of coding for these conditions in Germany. Second, our data did not include patients without sepsis, thus we cannot make any conclusions if ACSC occur with higher frequency compared to other acute medical conditions. Third, we cannot trace individual patient paths to understand which diagnostics and treatments patients with ACSC received prior to their hospitalization in the outpatient setting, as outpatient diagnoses are only available on quarterly basis in Germany.

## Conclusions

ACSC and infection-related ACSC are important contributors to rehospitalizations in sepsis survivors. This underlines to need for structured aftercare programs and interventions in these patients, particularly for ACSC risk groups which comprise older, care dependent patients in rural areas.

**Table 1:** Patient demographics and clinical features of sepsis hospital survivors

	*N* = 234,874	95% CI^1^
Demographics and comorbidity		
Age, mean (SD); median (Q1, Q3)	71.8 (14.1); 75 (64; 82)	
Sex, female, n (%)	107,474 (45.8%)	[45.6%, 46.0%]
Nursing care grade, n (%)		
0	128,951 (54.9%)	[54.7%, 55.1%]
1	4,284 (1.8%)	[1.6%, 2.0%]
2	37,783 (16.1%)	[15.9%, 16.3%]
3	32,050 (13.6%)	[13.4%, 13.9%]
4	21,650 (9.2%)	[9.0%, 9.4%]
5	10,156 (4.3%)	[4.1%, 4.5%]
Pre-existing nursing home residence, n (%)	30,210 (12.9%)	[12.7%, 13.0%]
Charlson Index (unweighted), mean (SD), median (Q1, Q3)	3.6 (2.3); 3 (2; 5)	
Diabetes, n (%)	107,532 (45.8%)	[45.6%, 46.0%]
Chronic heart failure or Myocardical infaction, n (%)	99,782 (42.5%)	[42.3%, 42.7%]
Chronic pulmonary disease, n (%)	80,614 (34.3%)	[34.1%, 34.5%]
Chronic renal disease, n (%)	91,335 (38.9%)	[38.7%, 39.1%]
Chronic liver disease, n (%)	46,674 (19.9%)	[19.7%, 20.0%]
**Acute sepsis**		
Septic shock, n(%)	22,464 (9.6%)	[9.4%, 9.7%]
Organ dysfunctions, n (%)		
Respiratory failure	119,480 (50.9%)	[50.7%, 51.1%]
Coagulopathy	39,343 (16.8%)	[16.6%, 16.9%]
Cardiovascular failure	99,267 (42.3%)	[42.1%, 42.5%]
Liver failure	3,884 (1.7%)	[1.6%, 1.7%]
Renal failure	59,057 (25.1%)	[25.0%, 25.3%]
Encephalopathy	57,644 (24.5%)	[24.4%, 24.7%]
Number of organ dysfunctions (mean (SD), median (Q1, Q3))	1.6 (1.0); 1 (1; 2)	
Focus of infection, n (%)		
Lower respiratory tract infection	92,664 (39.5%)	[39.3%, 39.7%]
Urinary system	87,540 (37.3%)	[37.1%, 37.5%]
Skin, wound, soft tissue	25,993 (11.1%)	[10.9%, 11.2%]
Intraabdominal, retroperitoneal	27,233 (11.6%)	[11.5%, 11.7%]
Intrathoracic	3,874 (1.6%)	[1.6%, 1.7%]
CNS	2,733 (1.2%)	[1.1%, 1.2%]
Bloodstream, vascular infections	19,256 (8.2%)	[8.1%, 8.3%]
Bone, joints	7,512 (3.2%)	[3.1%, 3.3%]
GI-tract, diarrhea	27,035 (11.5%)	[11.4%, 11.6%]
Systemic viral infections	3,933 (1.7%)	[1.6%, 1.7%]
Genital organs, sexually transmitted infections	4,376 (1.9%)	[1.8%, 1.9%]
Unspecific infections	140,999 (60.0%)	[59.8%, 60.2%]
Devise-related infections	18,404 (7.8%)	[7.7%, 7.9%]
Infection with seasonal Influenza	2,315 (1.0%)	[0.9%, 1.0%]
**Clinical features**		
Mechanical ventilation, n (%)	53,704 (22.9%)	[22.7%, 23.0%]
Renal replacement therapy, n (%)	17,846 (7.6%)	[7.5%, 7.7%]
Hospital length of stay (mean (SD); median (Q1, Q3))	22.8 (22.0); 16 (9; 29)	
ICU treatment, n (%)	86,996 (37.0%)	[36.8%, 37.2%]
Surgical treatment, n (%)	93,513 (39.8%)	[39.6%, 40.0%]
^1^ CI = Confidence Interval		

**Table 2:** Coefficients estimates of simple and multiple logistic regression for the outcome ACSC rehospitalization 12-months post-discharge

	simple Regression				multiple Regression			
Risk factor	Odds Ratio	95%CI		p-value	Odds Ratio	95%CI		p-value
Age (reference: <40)								
40–64 years	1.615	1.527	1.708	< 0.0001	1.560	1.475	1.651	< 0.0001
65–79 years	2.026	1.918	2.141	< 0.0001	1.857	1.757	1.963	< 0.0001
≥80 years	1.928	1.825	2.038	< 0.0001	1.645	1.555	1.740	< 0.0001
Gender (reference: female)								
male	1.151	1.132	1.171	< 0.0001	1.206	1.186	1.227	< 0.0001
Nursing care dependency (reference: no nursing care dependency)								
Nursing care dependency without nursing home	1.577	1.548	1.607	< 0.0001	1.589	1.558	1.620	< 0.0001
Nursing care dependency with nursing home	1.298	1.265	1.332	< 0.0001	1.313	1.278	1.349	< 0.0001
Place of residence (reference: urban)								
rural	1.033	1.014	1.053	0.0005	1.027	1.008	1.046	0.0061

## Data Availability

The authors confirm that the data utilized in this study cannot be made available in the manuscript, the supplemental files, or in a public repository due to German data protection laws (‘Bundesdatenschutzgesetz’, BDSG). Therefore, they are stored on a secure drive in the WIdO, to facilitate replication of the results. Generally, access to data of statutory health insurance funds for research purposes is possible only under the conditions defined in German Social Law (SGB V § 287). Requests for data access can be sent as a formal proposal specifying the recipient and purpose of the data transfer to the appropriate data protection agency. Access to the data used in this study can only be provided to external parties under the conditions of the cooperation contract of this research project and after written approval by the sickness fund. For assistance in obtaining access to the data, please contact wido@wido.bv.aok.de.
